# Knowledge Gaps and Barriers to Care in Men with Postprostatectomy Incontinence: Evidence from the German ProKontinenz Trial

**DOI:** 10.1016/j.euros.2026.04.014

**Published:** 2026-06-22

**Authors:** Viktoria Menzel, Christoph Kowalski, Sophie Klara Schellack, Rainer Koch, Sebastian Dieng, Axel Haferkamp, Alexander Winter, Andreas Blana, Carsten Schwarzer, Andreas Gonsior, Karl H. Tully, Stefan Baltes, Ingo Kausch, Andreas Manseck, Serdar Deger, Firas Jamour, Jörg Erdmann, Ina H. Kunz, Holger Kujau, Martina Schleder, Anna Schulze, Jan Ramm, Markus Straub, Jasper Koenig, Dennis Wielander, Andreas Neisius, Hans-Peter Gerbershagen, Felix Wezel, Stefan Conrad, Reinhard Hofmann, Simon Müller, Karl-Dietrich Sievert, Rishav Pradhan, Roger Riexinger, Petra Stamm, Thomas Knoll, Oliver Hahn, Johannes Graff, Ernst-Günther Carl, Johannes Huber, Christian Thomas, Martin Baunacke

**Affiliations:** a Department of Urology, Medical Faculty Carl Gustav Carus TU Dresden, Dresden, Germany; b Deutsche Krebsgesellschaft e.V., Berlin, Germany; c OnkoZert GmbH, Neu-Ulm, Germany; d Department of Urology, University Hospital Johannes Gutenberg, Mainz, Germany; e University Hospital for Urology, Klinikum Oldenburg, Department of Human Medicine, School of Medicine and Health Sciences, Carl von Ossietzky University Oldenburg, Oldenburg, Germany; f Department of Urology, Klinikum Fürth, Fürth, Germany; g Department of Urology, Klinikum Dortmund, Dortmund, Germany; h Department of Urology, University Hospital Leipzig AöR, Leipzig, Germany; i Department of Urology and Neurourology, Marien Hospital Herne, Ruhr-University Bochum, Herne, Germany; j KRH Klinikum Region Hannover, Klinikum Siloah, Hannover, Germany; k Department of Urology and Paediatric Urology, Ammerland Klinik GmbH, Westerstede, Germany; l Department of Urology, Klinikum Ingolstadt, Ingolstadt, Germany; m Department of Urology, Medius-Klinik Ostfildern-Ruit, Ostfildern, Germany; n Department of Urology, Klinikum Westmünsterland, Ahaus, Germany; o Prostate Cancer Centre Tauber-Franken, Bad Mergentheim, Germany; p Department of Urology, Zeisigwaldkliniken Bethanien, Chemnitz, Germany; q Department of Urology, SRH Wald-Klinikum Gera, Gera, Germany; r Department of Urology, St. Elisabeth Hospital Straubing, Straubing, Germany; s Department of Urology, Dresden Friedrichstadt General Hospital, Dresden, Germany; t Department of Urology, Sana Klinikum Leipziger Land, Borna, Germany; u Department of Urology, Klinikum Landshut, Region Landshut, Germany; v Department of Urology, KRH Klinikum Großburgwedel, Burgwedel, Germany; w Department of Urology, Vivantes Auguste-Viktoria Klinikum, Berlin, Germany; x Department of Urology, Brüderkrankenhaus Trier, Trier, Germany; y Department of Urology, Klinikum Ludwigsburg, Ludwigsburg, Germany; z Department of Urology, Ulm University Hospital, Ulm, Germany; aa Department of Urology, DIAKOVERE Friederikenstift, Hannover, Germany; bb Department of Urology, Klinikum Wolfsburg, Wolfsburg, Germany; cc Department of Urology, Klinikum Trauenstein/Kliniken Südostbayern AG, Trauenstein, Germany; dd Department of Urology, Medical School and University Medical Center OWL, Campus Klinikum Lippe, Bielefeld University, Detmold, Germany; ee Department of Urology, Katholische St. Lukas Gesellschaft mbH, St.-Josefs Hospital Dortmund, Dortmund, Germany; ff Department of Urology, Klinikverbund Südwest, Kreisklinikum Nagold, Nagold, Germany; gg Department of Urology, Heilig Geist-Krankenhaus Köln, Köln, Germany; hh Department of Urology, Sindelfingen-Boeblingen Medical Center, University of Tübingen, Sindelfingen, Germany; ii Department of Urology, University Hospital Würzburg, Würzburg, Germany; jj Department of Urology, St. Elisabeth-Krankenhaus Köln-Hohenlind, Köln, Germany; kk Bundesverband Prostatakrebs Selbsthilfe e.V., Bonn, Germany; ll Department of Urology, University Hospital Heidelberg, Heidelberg, Germany

**Keywords:** Postprostatectomy incontinence, Stress urinary incontinence, Continence care, Treatment barriers, Health information, Health service research

## Abstract

**Background and objective:**

Postprostatectomy incontinence (PPI) reduces quality of life, yet remains undertreated despite effective surgical options. Persistently low intervention rates in Europe for PPI suggest a care gap. This study assessed patients’ knowledge of surgical PPI treatments and barriers to treatment uptake.

**Methods:**

Cross-sectional baseline analysis of *ProKontinenz* trial. Men with persistent PPI for ≥12 mo after radical prostatectomy (≥2 pads/d, no prior incontinence surgery) from 34 certified prostate cancer centers were surveyed during January–June 2025, using validated questionnaires and 24-h pad test. The primary outcome was knowledge of incontinence surgery; secondary outcomes included information sources, treatment barriers, and associations with symptom burden/quality of life. Associations with knowledge were assessed using logistic regression.

**Key findings and limitations:**

A total of 526 of 692 men participated (90% response rate). Among the participants, common reasons for not considering incontinence surgery were satisfaction with incontinence products (79%), concerns about surgical risks (53%), and doubts about success of surgery (51%). Fifty-nine percent men reported no knowledge of surgical PPI treatment. Independent predictors of lacking knowledge were low urine loss (odds ratio [OR] 2.4, 95% confidence interval [CI] 1.3–4.7), less severe King’s Health Questionnaire (KHQ)-score “role limitation” (OR 2.2, 95% CI 1.1–4.3), concerns about treatment success (OR 2.2, 95% CI 1.2–4.1), and missing information from the urologist (OR 4.2, 95% CI 1.1–16.7) or partner (OR 2.0, 95% CI 1.2–3.8). Limitations of the study include self-reported data and a nonvalidated definition of “knowledge.”

**Conclusions and clinical implications:**

Among patients with PPI for ≥12 mo after radical prostatectomy, more than half did not have any knowledge of potentially effective surgery. Information deficits and symptom severity influence knowledge. Strengthening guideline-based information and clinician-patient communication may help close this gap.

## Take Home Message

Most men with postprostatectomy incontinence lack knowledge of surgical options. Missing information from physicians and partners and symptom severity influence knowledge. Improving structured, guideline-based patient information may reduce this care gap.

ADVANCING PRACTICE **What does this study add?**

This large, multicenter study identifies major information deficits as the primary barrier to surgical treatment in men with postprostatectomy incontinence. Symptom severity alone does not explain why most affected men remain uninformed; instead, lack of information from physicians and partners strongly predicts unawareness of effective therapies. By linking objective pad-test data, validated quality-of-life measures, and information pathways, this study explores the mechanisms behind the well-documented treatment gap. These insights highlight communication as a modifiable factor to improve timely, guideline-based continence care.

**Clinical Relevance**

Although severe postprostatectomy incontinence has become relatively uncommon in the contemporary prostate cancer care pathway, it remains a highly burdensome condition for affected men and can substantially impair quality of life. This study highlights that many patients with persistent incontinence are unaware of effective surgical treatment options, underscoring the need for systematic and timely counseling. Standardized, guideline-based information on all available treatment options may improve informed decision-making, treatment uptake, and ultimately the quality of life of prostate cancer survivors. Associate Editor: Roderick van den Bergh, MD, PhD.

**Patient Summary**

Many men who leak urine after prostate surgery do not know that effective surgical treatments exist. In our study, men were often unaware of options because they had not received clear information. Better communication could help more patients access treatments that can greatly improve continence and quality of life.

## 1 Introduction

Stress urinary incontinence following radical prostatectomy (RP) for prostate cancer remains a significant complication with far-reaching implications for patients’ quality of life [1], [2]. Reported prevalence rates of postprostatectomy incontinence (PPI) vary widely between 8–15%, depending on definition and follow-up duration [3], [4]. The European Association of Urology underscored the relevance of this issue in 2023 by launching the An Urge to Act initiative, highlighting urinary incontinence as a major and growing socioeconomic problem across Europe [5]. Although PPI accounts for only a fraction of incontinence cases, it represents a clinically important subgroup in prostate cancer follow-up.

Exact data on RP numbers across Europe are unavailable, yet national figures suggest a substantial burden. In Germany alone, approximately 25 000–27 000 RPs are performed each year [6], with comparable numbers reported from France [7]. Based on published PPI rates, this corresponds to approximately 2500–3000 new cases of persistent PPI each year in a country such as Germany [6], indicating that the number of affected men across Europe is substantial. These data emphasize the clinical and socioeconomic relevance of PPI.

Beyond individual suffering, PPI imposes considerable costs on health systems through long-term use of continence aids, repeated outpatient consultations, and productivity loss due to delayed return to work [8], [9]. In some analyses, the cumulative costs of PPI management exceed the initial cost of the prostatectomy itself [10]. With an aging population in Europe and a stable prostate cancer incidence, this economic burden is expected to increase further [11].

Despite the availability of effective conservative and surgical treatment options, surgical treatment of PPI remains markedly undertreated across Europe. Registry data demonstrate strikingly low surgical incontinence intervention rates: only 3.0% of affected men undergo surgery in Sweden [12], 2.8% in Austria [13], 2.7% in Germany [14], and 2.5% in the United Kingdom [15]. Moreover, international data mirror this discrepancy, with merely 3–6% of men receiving surgery for PPI [16], [17], [18].

A small number of studies suggest that limited communication between physicians and patients may represent the gap. Urologists often underestimate the impact of PPI on quality of life, while patients may be reluctant to raise this issue [19]. However, the exact mechanism driving this persistent undertreatment remains unclear.

The aim of this study is to examine patients’ knowledge of PPI treatment options and the barriers preventing therapy uptake within the ProKontinenz trial.

## 2 Methods

The ProKontinenz trial is a national, multicenter, randomized intervention study in Germany. This analysis uses baseline data collected prior to randomization for examining patients’ knowledge of PPI therapies and barriers to care.

This study is conducted in 34 certified prostate cancer centers across Germany that are part of the prospective Prostate Cancer Outcomes (PCO) study [20]. As part of the PCO study, these patients complete a questionnaire 1 yr after treatment initiation, which assesses continence among other domains. Eligible participants in this study were men with persistent PPI for ≥12 mo after RP, using ≥2 pads/d and without prior incontinence surgery. Patients were recruited whose RP had occurred at least 1 yr and at most 7 yr earlier.

As part of the ProKontinenz trial, eligible patients were given a questionnaire in January 2025. The questionnaire assessed sociodemographic characteristics, urinary symptoms, information-seeking behavior, and objective urine loss. Patients completed a 3-d pad test documenting 24-h urine loss. Knowledge of established surgical treatments for PPI (artificial urinary sphincter, sling surgery, and urethral balloons) was captured by asking patients to name at least one option (“knowledgeable”) or none (“not knowledgeable”). In addition, seven potential barriers to undergo surgery were assessed using nonvalidated, study-specific items. Validated instruments included the European Organization for Research and Treatment of Cancer Quality of Life Questionnaire Core 30 (EORTC QLQ-C30) [21], the Patient Health Questionnaire-4 (PHQ-4) for anxiety/depression [22], and the International Consultation on Incontinence Questionnaire-Urinary Incontinence Short Form (ICIQ-UI SF), as well as the King’s Health Questionnaire (KHQ) for urinary symptom burden [23], [24].

Continuous variables are reported as median (interquartile range [IQR]), as appropriate; categorical variables are reported as frequencies and percentages. Group comparisons were performed used chi-square test and t-test, as appropriate. Analyses were performed using complete-case analysis; missing data were not imputed. Multivariable analysis of independent predictors associated with no knowledge of incontinence surgery was performed. The following variables were selected for multivariable analysis: the KHQ subscales, the ICIQ-UI SF, and urine loss as factors affecting quality of life; the information sources surveyed; the three most frequently cited barriers to seeking treatment; age; net income; and embarrassment when discussing the issue with partners and urologists. In addition, an analysis was conducted to examine the independence of the constructs of quality of life and information-seeking behavior regarding knowledge of incontinence surgery. To show the independence of the domains among each other regarding patient knowledge, we developed several logit models by hierarchical backward model choice and examined the difference of information between the statistical optimal models by likelihood ratio tests. We used the same set of patients without missing value in all variables included in the optimal models for consistent multivariate analyses. Model performances were evaluated using receiver operating characteristic analyses and Hosmer-Lemeshow goodness-of-fit tests. Analyses were conducted using SAS version 9.4 (SAS Institute, Cary, NC, USA). Ethics approval was obtained (BO-EK-419092023). All patients provided written informed consent.

## 3 Results

### 3.1 Patient characteristics and baseline continence profile

Of 692 potentially eligible patients, 25 patients had died and 80 patients were excluded due to unmet inclusion criteria. A total of 587 patients consented to participate, of whom 526 completed the questionnaire relevant to this analysis, resulting in a 90% response rate (Supplementary Fig. 1).

Median age at surgery was 68 yr (IQR 64–72 yr) and at the time of the survey was 71 yr (IQR 67–75 yr), with a median interval of 3.4 yr (IQR 2.4–4.7 yr) between both. Histopathologically, 11% of men had a Gleason score of 6, 60% had a Gleason score of 7, and 29% a high-grade score of ≥8. Median pad use was 2.3 pads/d (IQR 2–3.3 pads/d), and median urine loss amounted to 83.3 ml/d (IQR 28–646 ml/d). The median ICIQ-UI SF score was 11 (IQR 9–14) (Table 1).

**Table 1:** Study population overview and associated quality-of-life measures

			No knowledge of incontinence surgeries (*n* = 315)	Knowledge of incontinence surgeries (*n* = 211)
Age at surgery (yr)		Median (IQR)	68 (64–72)	67 (62–71)
Age at questionnaire (yr)		Median (IQR)	71 (68–75)	70 (66–74)
Time between surgery and questionnaire (yr)		Median (IQR)	3.6 (2.4–4.7)	3.3 (2.2–4.7)
Marital status (*n* missing = 11)		Single	36 (12%)	22 (11%)
		Married	274 (88%)	183 (89%)
Monthly net income (EUR) (*n* missing = 15)		≤1500	35 (11%)	19 (9.5%)
		1500–4000	227 (73%)	155 (78%)
		>4000	49 (16%)	26 (13%)
PHQ-4 depression score (*n* missing = 45)		Median (IQR)	1 (0–4)	2 (0–4)
Average pad usage per day (*n* missing = 153)		Median (IQR)	2.3 (2–3)	2.8 (2–4)
Daily urinary leakage (ml/d) (*n* missing = 89)		Median (IQR)	66 (20–432)	131 (40–981)
ICIQ-UI SF (*n* missing = 20)		Median (IQR)	11 (8–14)	13 (10–15)
Global health (EORTC QLQ-C30) (*n* missing = 11)		Median (IQR)	5 (4–6)	5 (4–5)
Quality of life (EORTC QLQ-C30) (*n* missing = 9)		Median (IQR)	5 (4–6)	5 (4–5)
King’s Health Questionnaire (KHQ) subscales (0% = not affected at all – 100% = extremely affected) (%)	Median (IQR)	Role limitations (*n* missing = 27)	62.5 (50–75)	75 (50–87.5)
		Personal relationships (*n* missing = 35)	60 (40–80)	66.7 (46.7–80)
		Physical limitations (*n* missing = 18)	62.5 (50–75)	75 (50–87.5)
		Social limitations (*n* missing = 19)	50 (25–50)	50 (25–75)
		Emotions (*n* missing = 15)	41.7 (33.3–66.7)	50 (33.3–66.7)
		Sleep/energy (*n* missing = 17)	50 (37.5–62.5)	50 (37.5–75)
		Measures of severity (*n* missing = 24)	80 (70–90)	80 (75–90)

EORTC QLQ-C30 = European Organisation for Research and Treatment of Cancer Quality of Life Questionnaire Core 30; EUR = euro; ICIQ-UI SF = International Consultation on Incontinence Questionnaire-Urinary Incontinence Short Form; IQR = interquartile range; PHQ-4 = Patient Health Questionnaire-4.

### 3.2 Patient knowledge, information sources, and concerns regarding incontinence surgery

**Fig. 1:**
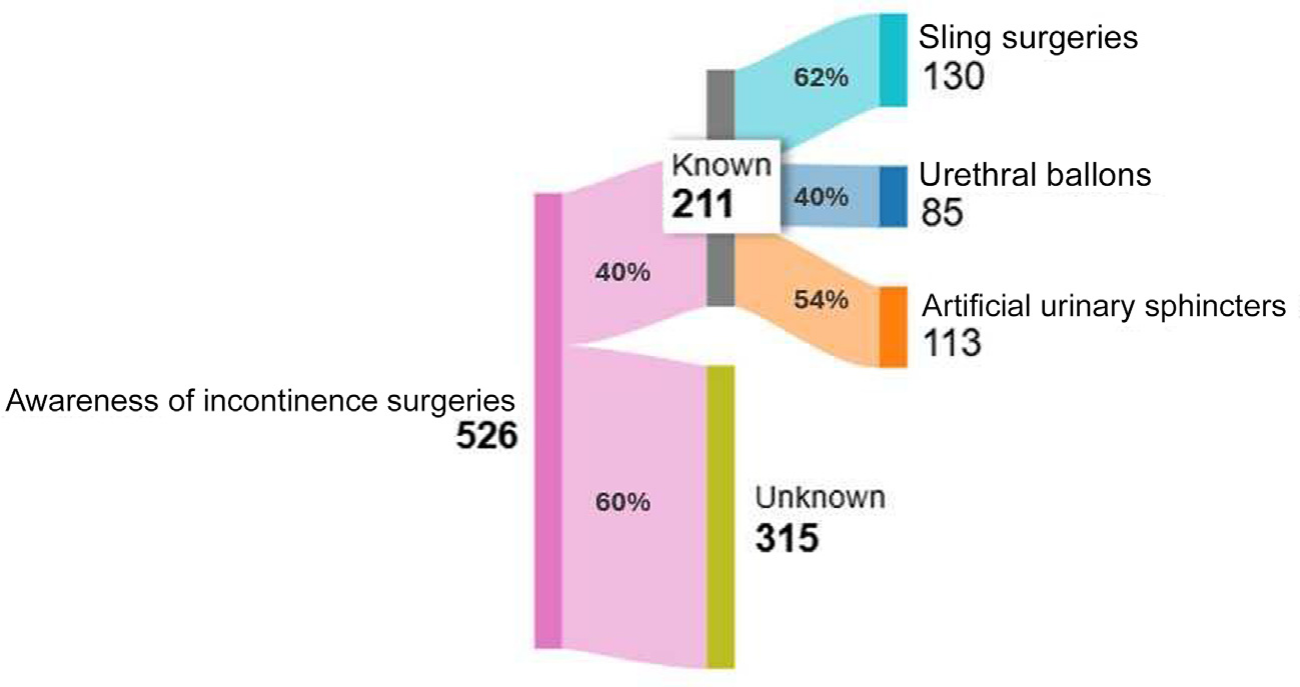
Knowledge of surgical therapies for urinary incontinence (*n* = 526). Categories are not mutually exclusive.

Of 526 respondents, 60% reported no prior knowledge of surgical treatment for PPI. Among the informed patients, 62% were familiar with male sling procedures, 54% with artificial urinary sphincters, and 40% with urethral balloons (Fig. 1).

The office urologist (92%) and the prostate surgery hospital (75%) were the most frequently cited information source regarding incontinence therapy, followed by the internet (68%) and partners (62%) (Table 2).

**Table 2:** Comparison of information-seeking behaviors in individuals with and without the knowledge of surgical therapies for incontinence

			No knowledge of incontinence surgeries (*n* = 315)	Knowledge of incontinence surgeries (*n* = 211)
Information sources and importance regarding continence therapy	Office urologist (*n* missing = 84)	No information	30 (11%)	6 (3.3%)
		Unimportant information	25 (10%)	16 (8.8%)
		Important information	207 (79%)	158 (88%)
	Another doctor (*n* missing = 165)	No information	87 (40%)	50 (35%)
		Unimportant information	39 (18%)	29 (20%)
		Important information	93 (42%)	63 (45%)
	Prostate surgery hospital (*n* missing = 145)	No information	66 (29%)	28 (18%)
		Unimportant information	31 (14%)	30 (19%)
		Important information	129 (57%)	97 (63%)
	Second opinion (*n* missing = 199)	No information	123 (62%)	64 (49%)
		Unimportant information	30 (15%)	21 (16%)
		Important information	44 (23%)	45 (35%)
	Support group (*n* missing = 209)	No information	152 (80%)	91 (72%)
		Unimportant information	30 (16%)	25 (20%)
		Important information	9 (4.7%)	10 (7.9%)
	Partner (*n* missing = 200)	No information	84 (42%)	40 (31%)
		Unimportant information	18 (9.0%)	27 (21%)
		Important information	97 (49%)	60 (48%)
	Relatives/friends (*n* missing = 201)	No information	103 (53%)	47 (36%)
		Unimportant information	49 (25%)	34 (26%)
		Important information	42 (22%)	50 (38%)
	Internet (*n* missing = 180)	No information	78 (38%)	31 (22%)
		Unimportant information	46 (23%)	49 (35%)
		Important information	80 (39%)	62 (43%)
	TV/radio (*n* missing = 203)	No information	126 (65%)	62 (48%)
		Unimportant information	50 (26%)	44 (34%)
		Important information	19 (9.7%)	22 (18%)
	Brochures/magazines (*n* missing = 181)	No information	85 (41%)	36 (26%)
		Unimportant information	54 (26%)	42 (31%)
		Important information	69 (33%)	59 (43%)
Frequency of internet use for health information (*n* missing = 9)		Daily	13 (4.1%)	11 (5.3%)
		At least once a week	52 (17%)	54 (26%)
		Less often	179 (57%)	116 (57%)
		Not at all	69 (22%)	23 (12%)
Discussion of incontinence with urologist in prostate cancer follow-up (*n* missing = 11)		Yes, my urologist asks me regularly	198 (63%)	139 (68%)
		Yes, my urologist asks me irregularly	39 (13%)	25 (12%)
		Yes, but I have to bring it up myself	63 (20%)	36 (18%)
		No, I wish my urologist would ask me about my incontinence	9 (2.9%)	3 (1.5%)
		No, I do not want to talk about it	3 (1.0%)	0 (0%)
Shame when talking about incontinence	Ashamed to talk with partner (*n* missing = 52)	Disagree	225 (78%)	150 (80%)
		Neither nor	21 (7.3%)	9 (4.8%)
		Agree	41 (15%)	28 (15%)
	Ashamed to talk with my urologist (*n* missing = 34)	Disagree	256 (86%)	181 (92%)
		Neither nor	14 (4.7%)	7 (3.6%)
		Agree	26 (8.8%)	8 (4.1%)

TV = television.

**Fig. 2:**
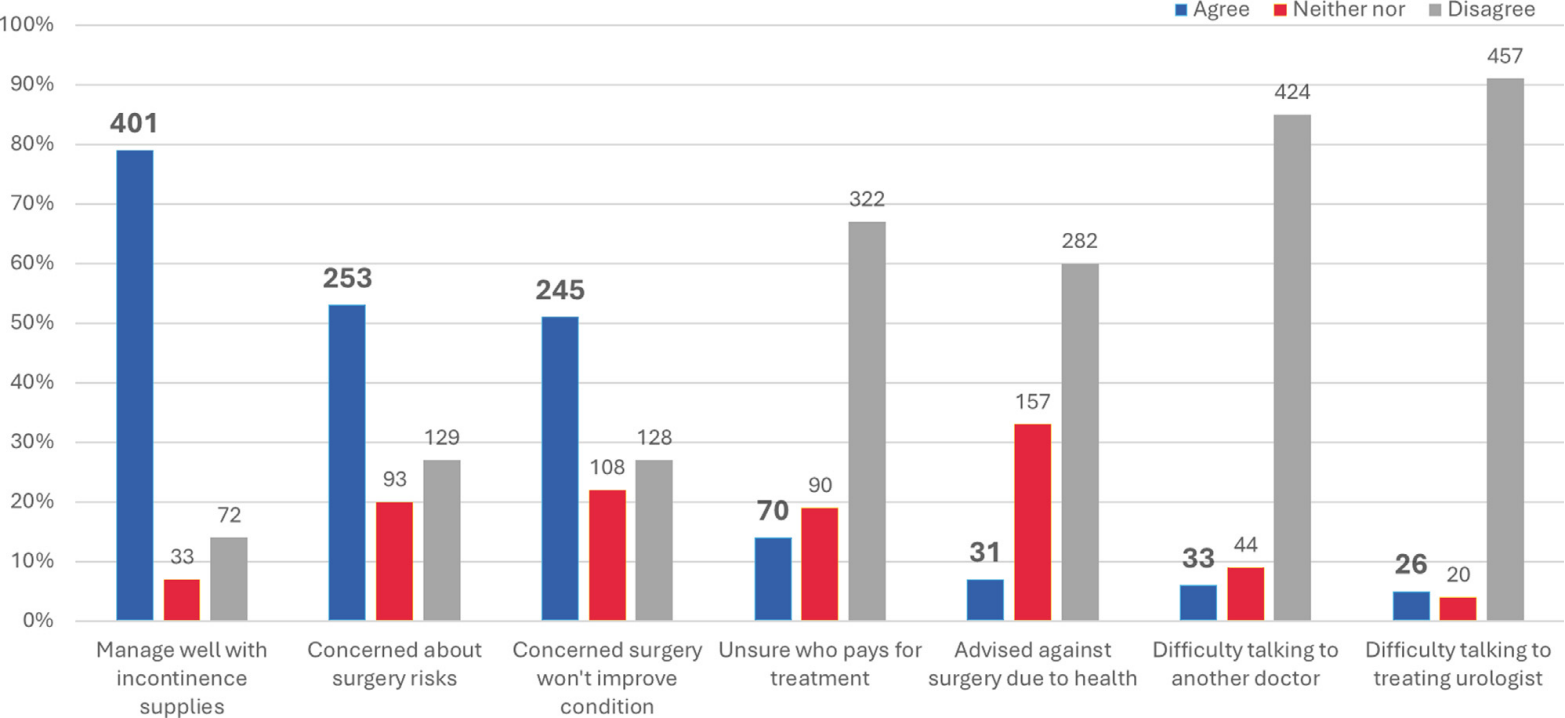
Patient barriers to seeking surgical incontinence treatment.

The most common reasons for not considering incontinence surgery were satisfactory management with incontinence products (79%), concerns about surgical risks (53%), and doubts about improvement of PPI condition after surgery (51%) (Fig. 2).

### 3.3 Predictors of limited surgical knowledge and its association with quality of life and information behavior

In the multivariable analysis, following parameters were predictors of lacking knowledge about incontinence therapy: low urine loss (odds ratio [OR] 2.4, 95% confidence interval [CI] 1.3–4.7, *p* = 0.008), less severe KHQ-score “role limitation” (OR 2.2, 95% CI 1.1–4.3, *p* = 0.02), concerns about treatment success (OR 2.2, 95% CI 1.2–4.1, *p* = 0.01), and missing information from the urologist (OR 4.2, 95% CI 1.1–16.7, *p* = 0.037) or partner (OR 2.0, 95% CI 1.2–3.8, *p* = 0.045) (Table 3).

**Table 3:** Multivariable analysis of independent predictors associated with no knowledge of incontinence surgery

Parameter	OR	*p* value
Low daily urinary leakage (<83.3 ml/d)	2.4 (1.3–4.7)	**0.008**
KHQ subscale “role limitation” (<62.5%)	2.2 (1.1–4.3)	**0.02**
Concerns about the success of therapy	2.2 (1.2–4.1)	**0.01**
No information from office urologist	4.2 (1.1–16.7)	**0.037**
No information from partner	2.0 (1.2–3.8)	**0.045**

KHQ = King’s Health Questionnaire; OR = odds ratio; bold = p<0.05

Analysis of the relationship between quality of life and information-seeking behavior shows that the domains are independent of one another, as evidenced by the quality-of-life variables (low daily urinary leakage [OR 3.1, 95% CI 1.6–5.9, *p* < 0.001] and managing incontinence well [OR 2.6, 95% CI 1.2–5.8, *p* = 0.01]) and the information-seeking behavior variables (lack of information from the hospital [OR 2.3, 95% CI 1.1–4.7, *p* = 0.03] and from the partner [OR 2.3, 95% CI 1.2–4.5, *p* = 0.02], as well as concerns about risks [OR 2.5, 95% CI 1.3–4.7, *p* = 0.004]) (Supplementary Table 1).

## 4 Discussion

In this multicenter study, PPI was associated with substantial functional burden. More than half of all men, who participated in this study, continued to experience relevant leakage several years after surgery, with quality-of-life scores indicating sustained physical and psychosocial impairment. Despite this burden, 60% had no knowledge regarding available incontinence therapies. Although most patients were able to cope with their symptoms (KHQ “severity measure”: 79%), concerns about treatment risks (53%) and success (51%) frequently constituted as major barriers. Predictors of lacking knowledge about incontinence therapy included low urine loss, low “role limitation” (the KHQ subscale), concerns regarding therapy success, and no information from the office urologist, the prostate surgery hospital, or the partner.

The cohort in this study includes men with PPI and reflects a selected population with a wide range of symptom severity and corresponding impact on quality of life. A major problem in PPI studies is the definition of urinary incontinence, as many rely solely on pad use and often define incontinence as >2 pads/d [3]. As a result, severity cannot be classified accurately. The Swedish LAPPRO study illustrates the distribution of pad use: among men using ≥2 pads/d, 76% reported 2–3 pads/d, which is comparable to the 2.3 pads/d in our cohort [25]. Few studies quantify PPI using pad tests, and their 1-h pad tests results correspond to our mean 24-h urine loss of 209 ml [26], [27]. Continence-specific quality of life measured using the ICIQ-UI SF is also rarely reported in PPI studies. The median ICIQ-UI SF score in our cohort was 11 (9–14), indicating moderate but clinically relevant impairment [28], [29], consistent with ICIQ-UI SF thresholds for moderate urinary incontinence reported in previous studies [30]. Despite this burden, those affected had not yet sought treatment for incontinence, even though more than 3.4 yr had passed since RP and guidelines recommend treatment after 1 yr of unsuccessful pelvic-floor training [31]. Braun et al similarly reported that affected men often remain untreated for several years, with a lifetime surgery rate of only 4.3–13% and a median delay of approximately 4 yr—an unnecessary postponement interpreted as a missed opportunity for individualized, patient-centered care [32].

The primary barrier to undergoing incontinence surgery was coping (79%). Viewed positively, this may reflect an expression of unrestricted quality of life and thus no perceived need for treatment. However, it cannot be ruled out that this attitude results from coping strategies developed to manage the condition [33], [34]. This may be due to a lack of knowledge [35], and is reflected in other relevant barriers, such as fear of risks (53%), concerns about treatment success (51%), and uncertainty regarding cost coverage (14%).

For this study, lack of knowledge about incontinence surgery was defined as unawareness of any surgical procedure. The analysis revealed a significant knowledge deficit among patients: 60% were unaware of any surgical options. Multivariate analysis revealed independent predictors of lacking knowledge: low symptom burden (low urinary leakage [OR 2.4] and good KHQ subscale “role limitation” [OR 2.2]), concerns regarding treatment success (OR 2.2), and lack of information sources (urologist [OR 4.2] and partner [OR 2.0]). The role of missing information is noteworthy, given that majority of patients reported their office urologist as an important source of information (83%) and that incontinence is discussed (97%). Shaw et al found that discussions with urologists were the most influential factor guiding the decision to undergo surgery, second only to the expectation of achieving dryness [36], highlighting the importance of clinician-patient communication in decision-making. One reason for the discrepancy between apparently adequate communication with the office urologist and persistent knowledge deficits may be differing perceptions of quality-of-life impairment. Two studies suggest that urologists often rate patients’ quality of life more favorably than the patients themselves [19], [37]. Consequently, treatment options and expected outcomes may not be discussed in sufficient depth, which aligns with the finding that concerns about poor treatment success were an independent predictor, although surgical interventions have long been shown to achieve good outcomes [38].

Ultimately, the question arises as to the context in which symptom burden and information behavior operate. We analyzed this question with several logit models by hierarchical backward model choice. A comparison of the models reveals the considerable independence of the two complexes. This emphasizes that it is not the symptom burden alone that determines whether patients seek information about treatments, but that the availability of information is an equally decisive determinant of whether patients are informed (Supplementary Table 1). Likewise, studies have shown that better-informed patients are more likely to seek health care [39], a relationship also confirmed specifically for urinary incontinence [40].

Several limitations warrant consideration. First, the data are based on self-reported symptoms and patient recall, which may introduce reporting bias; recall bias may have influenced the assessment of knowledge in either direction, and its magnitude cannot be determined. In addition, missing data in several variables resulted in smaller subgroup sizes, potentially contributing to an underrepresentation of patients with mild symptoms. Furthermore, the definition of lacking knowledge about incontinence surgery has not been formally validated. Knowledge was defined as awareness of at least one surgical option and did not assess depth or clinical applicability; consequently, patients classified as “knowledgeable” may still have lacked sufficient information for informed decision-making. Another limitation is recruitment at certified prostate cancer centers, which may limit generalizability to other care settings. Data on surgical technique and adjuvant radiotherapy were available but were not analyzed and may represent unmeasured confounders. This potential selection bias may have influenced the observed findings; however, its direction and magnitude remain uncertain. Our study is the first to examine the globally recognized gap in care for men with PPI in a real-world, multicenter cohort, linking validated quality-of-life data and objective measures of urinary incontinence with information levels and treatment barriers.

## 5 Conclusions

A supply deficit in the management of PPI in men is likely to exist in many European countries. Although health care systems differ across Europe and additional country-specific barriers may exist, there are no structural treatment barriers within the German health care system: all men with PPI have access to urological care, and established surgical procedures are fully reimbursed. This underscores that information deficits substantially influence the utilization of surgical treatment for PPI. The relevance of this issue should be investigated in other European countries as well, since similar dynamics are likely to play a considerable role. To address this deficit, office urologists should be encouraged to proactively inform affected men, and professional associations should make publicly accessible, guideline-based information on PPI and its available treatment. Such resources can offer medically accurate guidance for patients and serve as reference tools for clinicians. The development of such an information platform is also an objective of the ProKontinenz trial, which includes this cohort.

  ***Author contributions:*** Martin Baunacke had full access to all the data in the study and takes responsibility for the integrity of the data and the accuracy of the data analysis.

  *Study concept and design*: Menzel, Baunacke, Carl, Kowalski, Schellack, Dieng.

*Acquisition of data*: Dieng, Menzel, Baunacke, Thomas, Haferkamp, Winter, Blana, Schwarzer, Gonsior, Tully, Baltes, Kausch, Manseck, Deger, Jamour, Erdmann, Kunz, Kujau, Schleder, Schulze, Ramm, Straub, Koenig, Wielander, Neisius, Gerbershagen, Wezel, Conrad, Hofmann, Müller, Sievert, Pradhan, Riexinger, Stamm, Knoll, Hahn, Graff.

*Analysis and interpretation of data*: Menzel, Baunacke, Koch.

*Drafting of the manuscript*: Menzel, Baunacke, Koch, Kowalski, Schellack.

*Critical revision of the manuscript for important intellectual content*: all authors.

*Statistical analysis*: Koch, Menzel, Baunacke.

*Obtaining funding*: Baunacke, Huber.

*Administrative, technical, or material support*: Menzel, Baunacke, Thomas, Haferkamp, Winter, Blana, Schwarzer, Gonsior, Tully, Baltes, Kausch, Manseck, Deger, Jamour, Erdmann, Kunz, Kujau, Schleder, Schulze, Ramm, Straub, Koenig, Wielander, Neisius, Gerbershagen, Wezel, Conrad, Hofmann, Müller, Sievert, Pradhan, Riexinger, Stamm, Knoll, Hahn, Graff, Huber.

*Supervision*: Baunacke.

*Other* (specify): None.

  ***Financial disclosures:*** Martin Baunacke certifies that all conflicts of interest, including specific financial interests and relationships and affiliations relevant to the subject matter or materials discussed in the manuscript (eg, employment/affiliation, grants or funding, consultancies, honoraria, stock ownership or options, expert testimony, royalties, or patents filed, received, or pending), are the following: None.

  ***Funding/Support and role of the sponsor*:** The study was financed by the Innovation Fund (01VSF23023) of the Federal Joint Committee (G-BA), Germany.

  ***Declaration of generative AI and AI-assisted technologies in the manuscript preparation process*:** During the preparation of this work, the authors used DeepL for language editing. After using this tool, the authors reviewed and edited the content as needed and take full responsibility for the content of the published article.
