# Supplementary material for: Knowledge Gaps and Barriers to Care in Men with Postprostatectomy Incontinence: Evidence from the German ProKontinenz Trial
Source: Eur Urol Open Sci. 2026 Jun 22;90:33–41. doi: 10.1016/j.euros.2026.04.014 (PMC13316280; doi:10.1016/j.euros.2026.04.014)
Supplement: Supplementary Data 1 [file mmc1.docx]

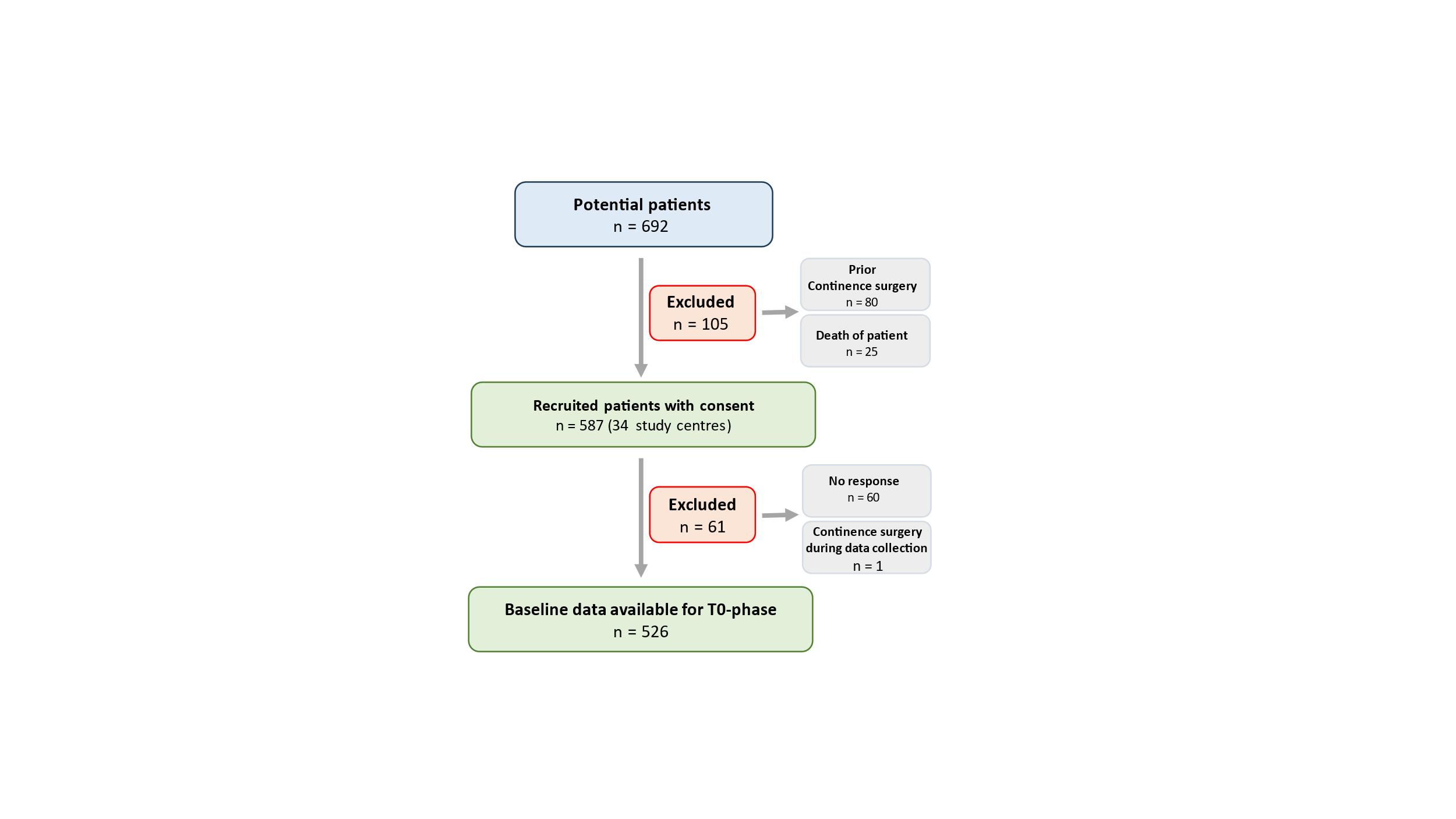


**Supplementary Figure 1:** CONSORT flow diagram of *ProKontinenz* trial.

| **Variable** | **Domain** | **OR (95%CI)**  **p value** | | | |
| --- | --- | --- | --- | --- | --- |
|  |  | **model with domain A**  **(1)** | **model with domains A and B**  **(2)** | **model with domains A and C**  **(3)** | **model with domains A and B and C**  **(4)** |
| Age at questionnaire  (<68 years) | A  **demographic** | 0.6 (0.4 - 1.1)  p=0.1 |  |  |  |
| Time between surgery and  Questionnaire (<3.4 years) |  |  |  |  | 0.6 (0.3 - 1.1)  p=0.1 |
| Low daily urinary leakage (<83.3ml/d) | B  **symptom/QOL** |  | 2.4 (1.3 - 4.4) **p=0.004** |  | 3.1 (1.6 - 5.9)  **p<0.001** |
| ICIQ-UI-SF (<11) |  |  | 1.9 (1.1 – 3.4) **p=0.02** |  |  |
| KHQ subscale “role limitation” (<62.5%) |  |  | 2.1 (1.1 - 3.9) **p=0.03** |  |  |
| Manage well with incontinence |  |  | 2.7 (1.4 - 5.3)  **p=0.005** |  | 2.6 (1.2 - 5.8)  **p=0.01** |
| No information from office urologist | C  **information-seeking behavior** |  |  | 3.5 (1.0 – 12.8)  p=0.06 | 3.3 (0.8 – 13.0)  p=0.09 |
| No information from prostate surgery hospital |  |  |  |  | 2.3 (1.1 – 4.7)  **p=0.03** |
| No information from partner |  |  |  | 1.8 (1.0 – 3.4)  p=0.06 | 2.3 (1.2 – 4.5)  **p=0.02** |
| No information from TV/Radio |  |  |  | 1.8 (1.0 – 3.3) p=0.06 |  |
| Concerned about surgery risks |  |  |  | 2.8 (1.5 – 5.0) **p<0.001** | 2.5 (1.3 – 4.7)  **p=0.004** |
| **Model characteristics** | | | | | |
| **AUC of the ROC** |  | 0.554 | 0.723 | 0.705 | 0.774 |
| **p value for Hosmer-Lemeshow goodness of fit test** |  | 0.1868 | 0.9554 | 0.9364 | 0.1868 |
| **-2ln(L)** |  | 335.527 | 285.094 | 272.061 | 239.980 |

**Supplementary Table 1**: Statistical optimal logistic regression models for several combinations of the domains continence-specific quality of life and information-seeking behavior. Number of used patients over all models: N=251 which 151 responses. Comparisons of models using likelihood ratio tests: (2) vs. (1): p<0.0001; (3) vs. (1): p<0.0001; (4) vs. (2): p<0.0001; (4) vs. (3): p<0.0001.
